# Supplementary material for: Sub-THz wireless transmission based on graphene-integrated optoelectronic mixer
Source: Nat Commun. 2023 Oct 13;14:6471. doi: 10.1038/s41467-023-42194-6 (PMC10575943; doi:10.1038/s41467-023-42194-6)
Supplement: Supplementary file 1 — Supplementary Information [file 41467_2023_42194_MOESM1_ESM.pdf]

Supplementary Information for "Sub-THz wireless transmission based on graphene integrated optoelectronic mixer"

A, Montanaro[1,2], G. Piccinin[3,4], V. Mišeikis[4,5], V. Sorianello[1], M.A. Giambra[6], S. Soresi[6], L. Giorgi[7], A. D'Errico[7], K. Watanabe[8], T. Taniguchi[9], S. Pezzini[10], C. Coletti[4], 5, and M. Romagnoli[1]

[1] *Photonic Networks and Technologies Lab – CNIT, Via G. Moruzzi,1 56124 - Pisa, Italy*
[2] *TeCIP Institute, Scuola Superiore Sant'Anna, via G. Moruzzi 1, 56124 Pisa, Italy*
[3] *NEST, Scuola Normale Superiore, Piazza San Silvestro 12, 56127 Pisa, Italy*
[4] *Center for Nanotechnology Innovation @NEST, Istituto Italiano di Tecnologia, Piazza San Silvestro 12, 56127 Pisa, Italy*
[5] *Graphene Labs, Istituto Italiano di Tecnologia, Via Morego 30, 16163 Genova, Italy*
[6] *Inphotec, CamGraPhIC srl, via G. Moruzzi 1, 56124 Pisa, Italy*
[7] *Ericsson Research, via G. Moruzzi 1, 56124 Pisa, Italy*
[8] *Research Center for Electronic and Optical Materials, National Institute for Materials Science, 1-1 Namiki, Tsukuba 305-0044, Japan*
[9] *International Center for Materials Nanoarchitectonics, National Institute for Materials Science, 1-1 Namiki, Tsukuba, 305-0044, Japan and*
[10] *NEST, Istituto Nanoscienze-CNR and Scuola Normale Superiore, , P.zza S. Silvestro 12, 56127 Pisa, Italy*

CONTENTS

I. Device design and simulation 2
A. Optoelectronic mixing 2
B. RF design and simulation 3
C. Optical absorption calculation 4
D. Conductivity calculation 5
E. Upconversion efficiency 7

II. Wireless link experimental setup 9

III. Conversion efficiency vs Optical Local oscillator power 10

IV. Error-Vector magnitude measurement: back-to-back 11

V. Supplementary Figures 12

Supplementary References 18

I. DEVICE DESIGN AND SIMULATION

A. Optoelectronic mixing

The sub-THz transmitter presented in the main text is based on a graphene optoelectronic mixer. The operating principle of the device relies on the possibility of modulating the electrical conductivity of a graphene layer by means of an optical field. Let's consider a graphene layer in dark conditions, with conductivity $\sigma_{dark} = 1/\rho_{dark}$, being ρ the resisitivity of the material. As detailed in I D, the coupling of the material with an optical field results in a change of the electronic temperature T_e and, consequently, of the chemical potential μ_c[1]. This translates in a change of the electrical conductivity[1], so that in illumination conditions one has:

$$\sigma_{light} = \sigma_{dark} + \Delta\sigma \tag{S.1}$$

Thus, the conductance of a graphene layer of length L and width W under homogeneous illumination is:

$$G_{light} = G_{dark} + \Delta G = \sigma_{dark}\frac{W}{L} + \Delta\sigma\frac{W}{L} \tag{S.2}$$

And the corresponding resistance is:

$$R_{light} = (\frac{1}{R_{dark}} + \frac{1}{\Delta R})^{-1} \tag{S.3}$$

Being $R_{dark} = 1/G_{dark}$ and $\Delta R = 1/\Delta G$. This corresponds to the parallel of two resistors. Supplementary Figure 1a takes up the scheme of the device presented in Fig. 3 of the main text. In our experiment, the graphene layer is embedded inside an electrical coplanar waveguide, which is a transmission line with characteristic impedance $Z_0 = 50\Omega$ (as detailed in I B), fed by a voltage generator V_{in} with output impedance $R_S = 50\Omega$, and connected to an antenna with impedance $R_L = 50\Omega$. The equivalent circuit of the graphene layer is deduced from equation S.3, as shown in the zoom of Supplementary Figure 1a, where the graphene-metal contact resistance R_C is also included. The resulting circuit is shown in Supplementary Figure 1b.The circuit model is purely resistive and describes the device including metal/graphene contact resistance. This model completely keeps the behavior of the device and is sufficient to describe the optoelectronic mixing operation in the considered frequency range ($<$ 100 GHz). The graphene intrinsic dynamics limiting the frequency response of our device would be observed at frequencies well above our experiment ($\sim$ 500 GHz) as very recently shown [2] and therefore is not included in the model. Besides this, the passive RF circuitry embedding the device could lead to bandwidth degradation if not well designed. As shown in Sec. B, the transmission line embedding our device is a coplanar waveguide designed to have a characteristic impedance of 50 Ω in the whole operating frequency range. Since the CPW characteristic impedance coincides with the output impedance of the voltage generator, it is not included in the lumped model.

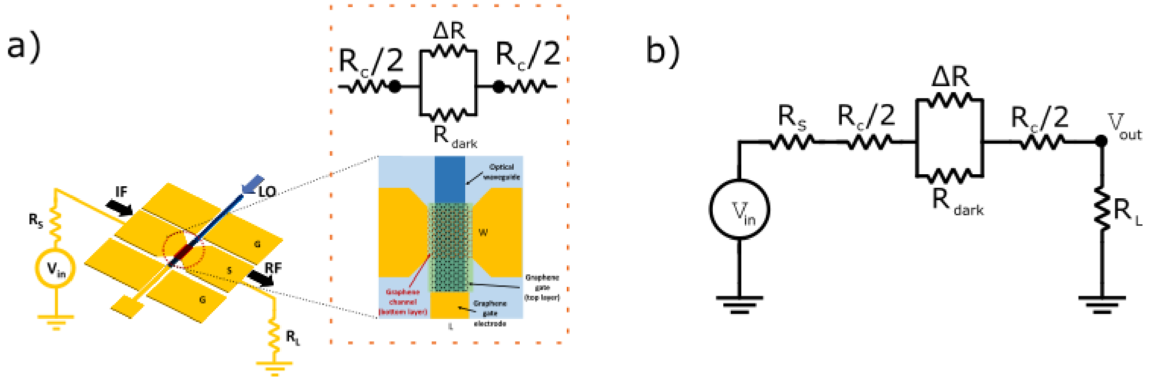

Supplementary Figure 1: Circuital model of the G-OEM. a) Circuital representation of a graphene resistor, with ΔR being the resistance contribution under light excitation. b) Circuital model of the graphene optoelectronic mixer including an input voltage generator, the contact resistance and the load resistor

By defining $R_A = R_S + R_C + R_L$, the output voltage V_{out} can be expressed as:

$$V_{out} = V_{in} R_L \underbrace{\frac{\Delta R}{R_A \Delta R + R_A R_{dark} + R_{dark} \Delta R}}_{①} + \underbrace{\frac{R_{dark}}{R_A \Delta R + R_A R_{dark} + R_{dark} \Delta R}}_{②} \tag{S.4}$$

The device acts as a frequency mixer when V_{in} and ΔR are time-varying. More specifically, let V_{in} be an electrical sinusoidal signal of frequency f_{ele}:

$$V_{in} = \widetilde{V_{in}} sin(2\pi f_{ele} t) \tag{S.5}$$

and ΔR be modulated at frequency f_{LO} by a time-varying optical signal. Thus:

$$\Delta R = \frac{1}{\Delta G} = \frac{L}{W} \frac{1}{\Delta\sigma} = \frac{L}{W} \frac{1}{\delta\sigma + \delta\sigma sin(2\pi f_{LO} t)} \tag{S.6}$$

Where the constant level $\delta\sigma$ accounts for the conductivity change induced by the mean optical power. To analytically visualize the mixing product, ① in S.4 can be approximated as:

$$① = \frac{\Delta R}{R_A \Delta R + R_A R_{dark} + R_{dark} \Delta R} \sim \frac{1}{R_A + R_{dark}}, \Delta R \gg R_A, R_{dark} \tag{S.7}$$

while ② can be approximated as:

$$② = \frac{R_{dark}}{R_A \Delta R + R_A R_{dark} + R_{dark} \Delta R} \sim \frac{1}{\Delta R} \frac{R_{dark}}{R_A + R_{dark}}, \Delta R \gg R_A, R_{dark} \tag{S.8}$$

Thus V_{out} can be written as:

$$V_{out} \sim \frac{\widetilde{V_{in}} sin(2\pi f_{ele} t) R_L}{R_A + R_{dark}} + \frac{\widetilde{V_{in}} sin(2\pi f_{ele} t) R_L R_{dark}}{R_A + R_{dark}} \frac{W}{L} (\delta\sigma + \delta\sigma sin(2\pi f_{LO} t)) \tag{S.9}$$

the second term in S.9 contains the product between the two time-varying signals, which gives:

$$\widetilde{V_{in}} sin(2\pi f_{ele} t) \delta\sigma sin(2\pi f_{LO} t) = \frac{\widetilde{V_{in}} \delta\sigma}{2} [cos(2\pi |f_{ele} - f_{LO}| t) + cos(2\pi (f_{ele} + f_{LO}) t)] \tag{S.10}$$

We used the G-OEM to implement the transmitting part of the wireless link. Nevertheless, from S.9 it follows that the G-OEM can also be used as down-converter in a sub-THz receiver. To evaluate the conversion efficiency of the G-OEM, we solved the circuit represented in Supplementary Figure 1b using equation S.4, that is, without using the approximations in equations S.7 - S.9. Moreover since the optical power coupled to the graphene layer is not homogeneously distributed along the channel (see I C, and I D), we calculated the conductance G_{dark} and G_{light} by integrating the spatial-dependent conductivity $\sigma(x, y)$ derived from simulations, as detailed in section I D.
It is worth noting that this device does not need any DC bias voltage applied to the graphene channel, when operated as optoelectronic mixer. This is a substantial difference compared to graphene-based photobolometers operating as photodetector, which are affected by high power consumption due to the need of DC current.

B. RF design and simulation

With reference to Supplementary Figure 1a-b, to match the output impedance of the voltage generator $R_S = 50\Omega$ and the output impedance of the load $R_L = 50\Omega$ represented by the antenna in the transmission system, the coplanar waveguide (CPW) embedding the graphene layer has been designed to have a characteristic impedance $Z_0 = 50\Omega$. Supplementary Figure 2 shows the top view of the CPW, and its cross section along the cut line indicated in red. The quasi-TEM electromagnetic mode is simulated using a commercial software (*Comsol Multiphysics*). The target impedance has been obtained for $S = 74\mu m$ and $gap = 17.5\mu m$.

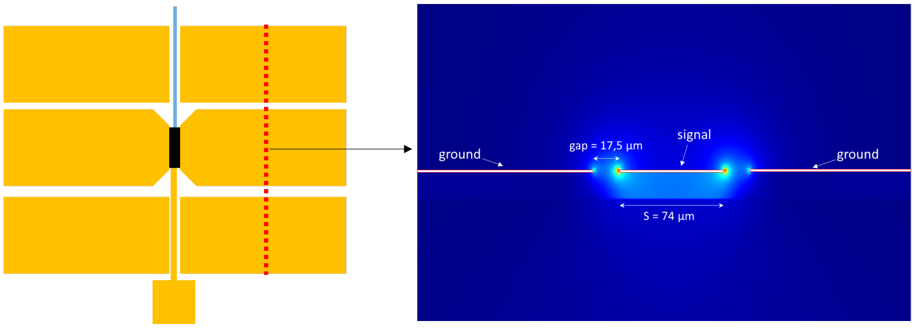

Supplementary Figure 2: CPW Simulation and design. Top view of the CPW and its cross-section with the quasi-TEM electromagnetic mode, at the level of the red cut line. The device has been fabricated using the dimensions indicated in the figure, which give a characteristic impedance $Z_0 \sim 50\Omega$

C. Optical absorption calculation

Light absorption allowing optoelectronic mixing takes place at the level of the bottom graphene active layer. Nevertheless, a fraction of the optical power is absorbed by the top graphene layer and by the metal contacts. This portion represents the insertion loss of the device. To evaluate the actual optical power that is absorbed by the graphene bottom layer (that is, the portion of power responsible of the graphene conductivity change), we adopted the same simulation procedure used in the *Supplementary Information* of [3]: we simulated the TE waveguide mode profile of the waveguide coupled to the active graphene layer. We then calculated the optical absorption per unit length $\alpha_{active\,layer}$ from the imaginary part of the effective refractive index. We then simulated the whole structure, comprising the top graphene and metal contacts, and extracted again the optical absorption per unit length α_{total}. By defining:

$$\eta = \frac{\alpha_{active\,layer}}{\alpha_{total}} \tag{S.11}$$

The absorbed power in the active layer along the optical mode propagation direction is[3]:

$$P(x,y) = \eta \frac{P_{in}}{\int_{-\infty}^{+\infty} \Re(\mathcal{P}_y(x, y=0, z=0))\,dx} \Re(\frac{1}{2}\mathcal{P}_y(x, y=0, z=0)) \frac{exp(\frac{y}{L_a})}{L_a} \tag{S.12}$$

where P_{in} is the input optical power, $\mathcal{P}_y$ is the Pointing vector component along the propagation direction, L_a is the absorption length, defined by:

$$-\frac{dP_{opt}(y)}{dy} = \frac{1}{L_a} P_{in} exp(\frac{y}{L_a}) \tag{S.13}$$

Being $P_{opt}(y)$ the optical power propagating along the structure at the coordinate y, undergoing exponential decay due to absorption[3, 4]:

$$P_{opt}(y) = P_{in} exp(\frac{y}{-L_a}) \tag{S.14}$$

The absorbed power in the active layer $P(x,y)$ is then used as heat source to calculate the electronic temperature along the graphene channel, as detailed in I D. The profile of $P(x,y)$ is shown in Supplementary Figure 3. The absorbed power density scale refers to an input power of $1mW$.

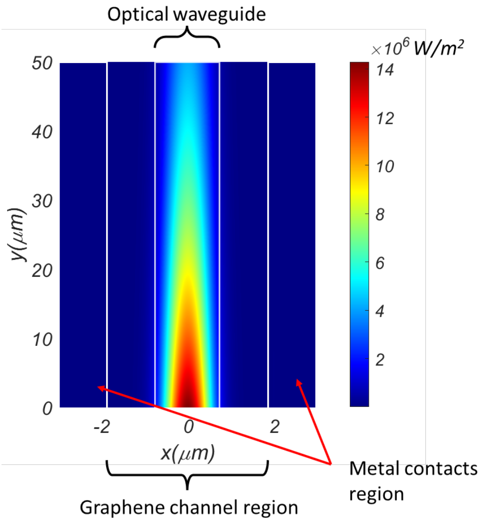

Supplementary Figure 3: G-OEM optical power absorption simulation. Simulated absorbed optical power density, in the active graphene layer (for an input power of 1 mW), which acts as heat source for hot electrons. The waveguide region is evidenced in white, as well as the metal contacts regions, between which the graphene active layer is present

D. Conductivity calculation

As discussed in I A, the G-OEM working principle is based on the change in the electrical conductivity of the graphene layer under optical excitation. The electrical conductivity can be expressed as[1, 5]:

$$\sigma(\omega, T_e, \mu_c) = \frac{D(\mu_c, T_e)}{\pi(\Gamma(\mu_c, T_e) - i\omega)} \tag{S.15}$$

where ω is the angluar frequency at which electrons drift in the material according to the Drude transport picture, μ_c the chemical potential, T_e is the electronic temperature, Γ the transport scattering rate and D the Drude weight. For a two-dimensional Dirac Fermions gas, this last reads [1]:

$$D(T_e) = \frac{2e^2}{\hbar^2} k_B T_e ln[2cosh(\frac{\mu_c(T_e)}{2k_B T_e})] \tag{S.16}$$

Where e is the elementary charge, $\hbar$ is the reduced Planck constant, k_B the Boltzmann constan. The expression of Γ in S.15 depends on the microscopic physical mechanisms limiting charges transport, which changes depending on graphene quality and on its electrostatic environment [6, 7]. We phenomenologically model Γ assuming constant mobility μ as a function of the carriers chemical potential μ_c. In this case, the transport scattering rate can be deduced from [8]:

$$\Gamma(\mu_c) = \frac{1}{\tau} = \frac{ev_F^2}{\mu_c \mu} \tag{S.17}$$

The angular frequency in the sub-THz range is $\sim 10^{11} rad/s$, while Γ lies in the range $\sim 10^{12} - 10^{13} rad/s$ [9]. We thus neglect the frequency dependence in equation S.15. The optical power coupled to the grpahene channel induces a change in T_e and in μ_c so that the change in conductivity $\Delta\sigma$ in equation S.1 can be expressed as:

$$\Delta\sigma = \sigma_{dark} - \sigma_{light} = \frac{D(\mu_c(T_{e,room}), T_{e,room})}{\pi(\Gamma(\mu_c(T_{e,room}), T_{e,room}))} - \frac{D(\mu_c(T_{e,hot}), T_{e,hot})}{\pi(\Gamma(\mu_c(T_{e,hot}), T_{e,hot}))} \tag{S.18}$$

$T_{e,room}$ is the electrons temperature in dark conditions, which coincides with the lattice temperature. $T_{e,hot}$ is the hot electrons temperature due to light coupling. The temperature dependence of the chemical potential $\mu_c(T_e)$ is found by numerical inversion of the charge carriers conservation formula [10]:

$$\frac{2}{\pi} \frac{(k_B T)^2}{(\hbar v_F)^2} [Li_2(-e^{\frac{-\mu_c}{k_B T}}) - Li_2(-e^{\frac{\mu_c}{k_B T}})] = \frac{C_{ox} V_{GS}}{e} - \frac{\alpha \mu_c C_{ox}}{e^2} \tag{S.19}$$

Being v_F the Fermi velocity, Li_2, the dilogarithm function [10], V_{GS} the top gate voltage. α is 1 or 2, for respectively, metallic or graphene gates [10, 11]. In our case, $\alpha = 2$. $C_{ox} = \frac{\epsilon\epsilon_{hBN}}{t_{hBN}}$ is the geometrical capacitance, which depends on the hBN dielectric constant ϵ_{hBN} and on its thickness t_{hBN}. ϵ is the vacuum permittivity. We then account for the effect of electrons-holes puddles by substituting the chemical potential found in equation S.19 with[1]:

$$\mu \rightarrow \sqrt[4]{\mu_c^4 + \mu_{puddles}^4} \tag{S.20}$$

Where $\mu_{puddles}$ is calculated from the charge inhomogeneity n_0 at the charge neutrality point [10]:

$$\mu_{puddles} = \sqrt{n_0 \pi} \hbar v_F \tag{S.21}$$

In our sample, $\mu_{puddles} \sim 0.033 eV$, since $n_0 \sim 8 \cdot 10^{10}$ (see main text). To get the spatial profile of the electronic temperature under optical excitation, we solved the heat equation. For high mobility samples this reads [5]:

$$-\nabla \cdot (\mathcal{L}\sigma T_e \nabla T_e) + \frac{C_e}{\tau_{cool}}(T_e - T_{ph}) = P(x, y) \tag{S.22}$$

The left hand of equation S.22 contains two cooling terms: the first is related to the electronic heat conduction $\mathcal{L}\sigma T_e$, defined by the Wiedeman-Franz law, where $\mathcal{L} = \frac{\pi^2 k_B^2}{3e^2}$ is the Lorenz number, σ is the electrical conductivity. The second term $\mathcal{Q} = \frac{C_e}{\tau_{cool}}(T_e - T_{ph})$ is the energy transfer rate associated to the hyperbolic phonon polariton radiative cooling occourring in hBN-encapsulated graphene samples [12]. T_{ph} is the phonon temperature. We assume $T_{ph} = T_{room} = 300K$. C_e is the specific heat of graphene. This quantity is usually approximated, depending on the operating conditions. Specifically[5]:

$$\begin{aligned} C_e &= \frac{2\pi\mu_c}{3(\hbar v_F)^2} k_B^2 T_e \ for \ \frac{EF}{k_B T_e} \gg 1 \\ C_e &= \frac{18\zeta(3)}{\pi(\hbar v_F)^2} k_B^3 T_e^2 \ for \ \frac{EF}{k_B T_e} \ll 1 \end{aligned} \tag{S.23}$$

Being ζ the Riemann zeta function. In our operating conditions $\frac{E_F}{k_B T_e}$ is comparable to 1. Putting the first formula of S.23 in eq. S.22 overestimates the electrons temperature, while the use of the second formula of S.23 leads to an underestimation of the electrons temperature far from the charge neutrality point (G-OEM operating condition). We thus explicitly calculate the specific heat using the general formula [13]:

$$C_e = \int_{-\infty}^{+\infty} (E - E_F) \frac{df}{dT_e} g(E) \tag{S.24}$$

Where E is the energy of each charge particle, $E_F = \mu_c(T = 0K)$ is the Fermi level, f is the Fermi-Dirac distribution and $g(E)\frac{2}{\pi\hbar^2 v_F^2}|E|$ is the density of states in graphene [10]. In [14] authors calculated $\tau_{cool} \sim 2ps$ for $T_{ph} = 300K$ in high doping, weak-heating regime ($\mu_c \gg k_B T$, $T_e \sim T_{ph}$). In our experiment T_e can be significantly higher than T_{ph}, since we attain optical powers inducing T_e approaching $1000K$. Nevertheless, the same reference ([14]) compares the calculation in weak-heating regime with experimental data obtained in strong heating regime ($T_e \gg T_{ph}$), revealing very small deviation from the two conditions. Based on these considerations, we used $\tau_{cool} \sim 2ps$. The right hand of equation S.22 is the heat source, represented by the optical power absorbed along the graphene channel, defined by equation S.12. S.22 does not include the supercollisions cooling term [15], as it is predominant only in low mobility samples [9, 15]. We obtained the temperature profile along the graphene channel using a finite-element commercial solver (*Comsol Multiphysics*), and consequently extracted the conductivity in light and dark conditions using S.15. Supplementary Figure 4a shows the electronic temperature profile Te, while Supplementary Figure 4b shows the chemical potential $\mu_c(Te)$ along the graphene channel, at a gate Voltage $V_G - V_{CNP} = 2.3V$, for a power input of 20 mW. These two quantities were then used to extract $\Delta\sigma$, as defined in S.18. This last is shown in Supplementary Figure 4c.

Because of the spatial dependence of $\sigma_{light}, \sigma_{dark}, \Delta\sigma$, the values G_{light} and G_{dark} (defined in S.3 in the case of homogeneous illumination) where actually calculated by numerical integration over the space coordinates:

$$G = \left[\int_0^L \frac{1}{\int_0^W \sigma(x, y) dy} dx \right]^{-1} \tag{S.25}$$

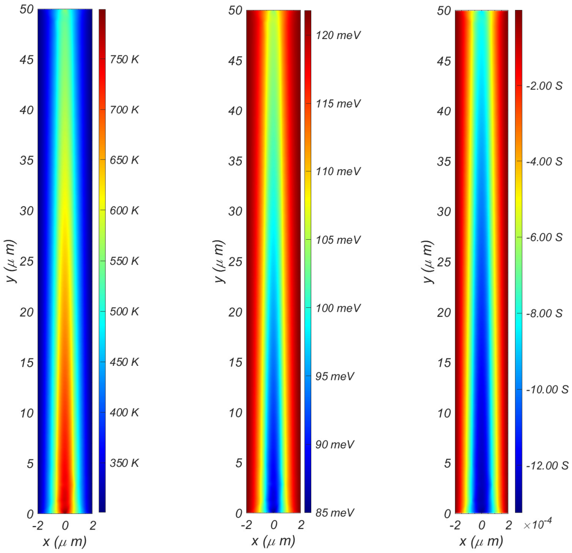

(a) (b) (c)

Supplementary Figure 4: Device Simulation. spatial map of: (a) Temperature, (b) Chemical potential(c) $\Delta\sigma$. The simulation is performed for an input optical power of 20 mW, $V_G - V_{CNP} = 2.3V$.

where $L = 4\mu m$ is the length of the graphene channel (electrical transport direction) and $W = 50\mu m$ is the width. The gate dependent change in conductivity under illumination $\Delta G(V)$ is shown in Supplementary Figure 5. The simulation well reproduces the typical photoconductivity curve of a biased graphene detector [16]: near the charge neutrality point, the material behaves similarly to a typical semiconductor (photoconductive regime) [1, 17] while at higher doping a conductivity decrease takes place due to carrier heating, analogously to metals (photobolometric regime) [1, 17].

E. Upconversion efficiency

The upconversion efficiency of the G-OEM was calculated in the following way: we first solved the circuit in Supplementary Figure 1. $\widetilde{V_{in}}$ in equation S.5 was set to $632.36mV$. This is the voltage applied by a power generator delivering a maximum power $P_{in} = 0dBm$ (1 mW) in matched conditions [18], i.e. when a 50Ω load is directly connected to the generator. We then calculated V_{out} from S.4, with ΔR defined by equation S.6. Finally, we computed the power spectral density of V_{out} across the load resistor $R_L = 50\Omega$:

$$\boldsymbol{P_{out}(f)} = \mathfrak{F}^2\left[\frac{V_{out}(t)}{R_L}\right] \tag{S.26}$$

Being $\mathfrak{F}$ the Fourier transform operator. We extracted the Fourier component at $f_{LO} + f_{ele}$, that is the upconverted power $P_{out} = \boldsymbol{P_{out}(f_{LO} + f_{ele})}$. We define the upconversion efficiency as:

$$P_{conv[dB]} = P_{out[dBm]} - P_{in[dBm]} \tag{S.27}$$

In the calculation we used an input power $P_{in} = 0dBm$, thus $P_{conv[dB]} = P_{out[dBm]}$. Supplementary Figure 6 shows the plot of the simulated upconversion efficiency for an input optical power of 20 mW, as a function of V_G. The conversion efficiency has a maximum of $41dB$ in the bolometric regime (high doping) for $V_G - V_{CNP} = 1.4$. This gate voltage defines the operating point of the G-OEM. We experimentally found an optimal EVM for $VG - V_{CNP} = 2.3V$, with upconversion efficiency of $\sim 44dB$. The difference between simulation and experiment can be attributed to non-idealities that are not taken into account in the simulation, e.g. the room temperature set to 300K, and Joule heating induced by the electrical power which actually changes the electrons and phonons rooom temperature. From simulations, we get the best upconversion efficiency at the charge neautrality point, while in our experiment we got poor EVM for $V_G = V_{CNP}$. This can be attributed to higher electrical reflections compared to the high doping

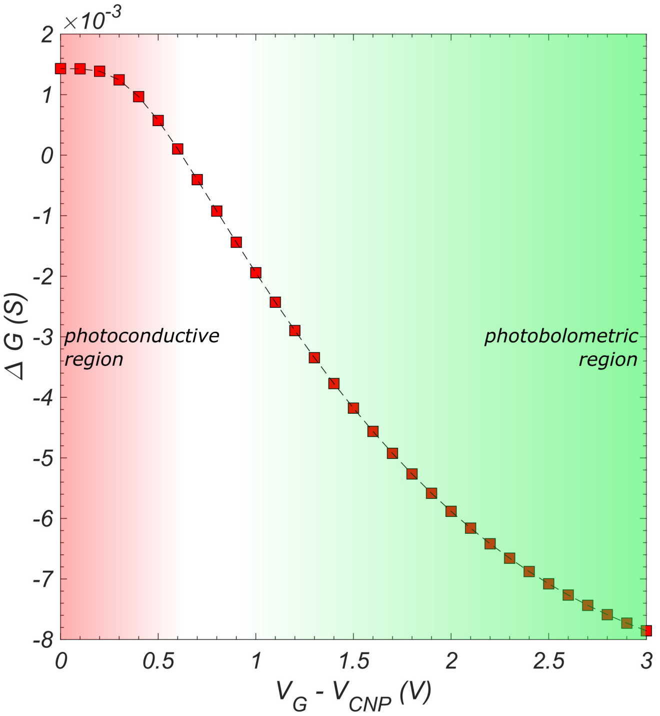

Supplementary Figure 5: Simulated graphene channel change in conductance (ΔG) under illumination, vs gate voltage. The simulation is performed for an input optical power of 20 mW.

operation, due to the high impedance mismatch between the 50Ω generator and the circuit when the graphene electrostatic doping is low.

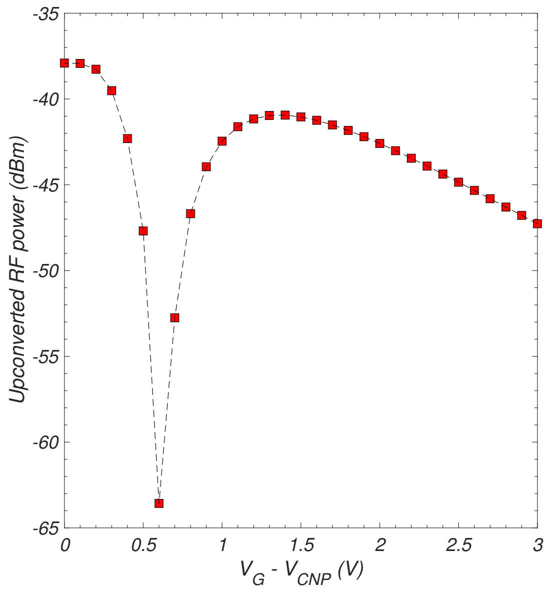

Supplementary Figure 6: Simulated upconversion efficiency vs Gate voltage, for an input optical power of 20 mW

To increase the wireless link distance and datarate (i.e., larger bandwidth and higher order modulation formats), the signal-to-noise ratio (SNR) has to be optimized. In the main text, we indicate some optimization for the wireless

link system, which are the use of higher gain antennas, more performing digital-to-analog converters (also discussed in IV) and lower noise electronics at both the receiver and the transmitter level. Besides system optimization, the SNR can be improved acting on the design of the G-OEM itself. The parameter to be optimized is the up-conversion efficiency, which is related to the device geometry and to the graphene quality. Concerning geometry, our design choice was tailored considering a contact resistance value in the range $1 - 2K\Omega\mu m$. If the contact resistance is lower, it is convenient to reduce the active area footprint. Considering a contact resistance of $\sim 500\Omega\mu m$, achievable at the wafer scale level (although currently only achievable at high carrier concentration level compared to the operating point of our G-OEM) [19], the graphene channel length and width can be reduced from $L = 4\mu m$ to $L = 2\mu m$ and from $W = 50\mu m$ to $W = 25\mu m$. We simulated this geometry using a uniformly distributed absorbed optical power along the channel. This condition can be achieved e.g. by coupling light from both sides of the waveguide underneath the graphene channel. In this condition, we obtain $P_{conv} \sim -33dB$, with performance boost of $\sim 11dB$ compared to the experimental value obtained with the present design. A further improvement could be obtained using plasmonic enhancement, which greatly improves the internal photoresponsivity [20].
Concerning graphene quality, we used hBN-encapsulated graphene to suppress super-collisions cooling [15] and obtain higher hot electrons temperatures and consequently higher responsivities compared to low mobility samples. The change in conductivity of graphene photobolometers is fundamentally due to a reduction of impurity and lattice disorder screening while rising the electronic temperature [9], which induces a mobility reduction, thus a conductivity decrease. To quantitatively evaluate the performance of a G-OEM based on low mobility graphene, we simulated the device using a mobility of 4000 $cm^2V^{-1}s^{-1}$, with charge carrier inhomogeneity $n_0 = 6 \cdot 10^{11}cm^{-2}$. These are typical values of graphene on SiO_2 [9]. In this conditions, long-range Coulomb scattering dominates transport, and supercollisions are the major pathway for hot electrons cooling [15]. We thus included this contribution in the heat equation and removed the hyperbolic phonon polariton radiative cooling term which only takes place in hBN-encapsulated graphene [12, 14]. In this case, the heat equation reads [15]:

$$-\nabla \cdot (\mathcal{L}\sigma T_e \nabla T_e) + A(T_e^3 - T_{ph}^3) = P(x, y) \tag{S.28}$$

Being A $= 9.62\frac{4g(\mu_c)}{\hbar k_F l}k_B^3$ [15, 21], , with k_F the Fermi wave vector. The result is shown in Supplementary Figure 7. The upconversion efficiency is ~ -55 dB in the photobolometric region, which is > 25 fold decrease compared to the simulation using high mobility graphene and the current design. The dependence of carriers mobility against temperature in low mobility samples, in which the transport is dominated by long-range Coulomb scattering, has been widely studied both theoretically and experimentally [9]. In hBN-encapsulated high mobility graphene, the dominant cooling pathways are hyperbolic phonon polariton radiative cooling and electronic heat conduction [12, 14], while transport is dominated by random strain disorder [6, 7]. This one has two contributions, i.e., random scalar potential and random gauge potential [6, 7]. The first contribution leads to temperature-dependent mobility, while the second doesn't [6, 7, 22]. Therefore, even if a further increase of mobility could lead to higher hot electrons temperatures, this may not correspond to a net enhancement of conductivity change. Future experimental studies on ultra-high mobility graphene bolometers could clarify this.

II. WIRELESS LINK EXPERIMENTAL SETUP

The experimental setup of the wireless link is shown in Supplementary Figure 8. The TX is composed by the G-OEM performing upconversion, a sub-THz amplifier and a TX antenna. The RX is composed by a receiving antenna and a commercial downconverter. The two horn antennas are 2-m far from each other.

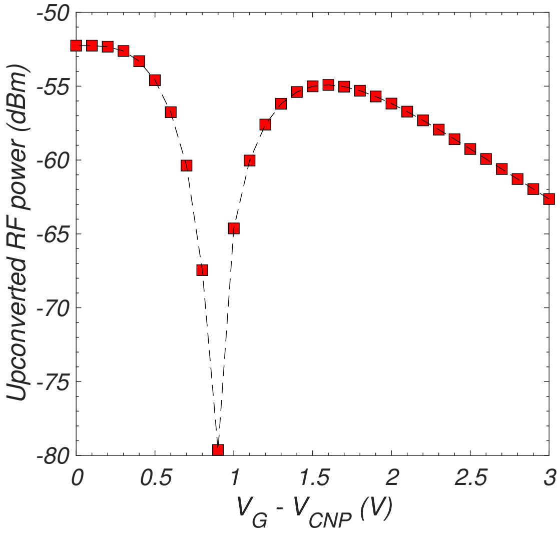

Supplementary Figure 7: Simulated upconversion efficiency vs Gate voltage for low mobility sample ($\mu = 4000 cm^2 V^{-1} s^{-1}$, $n_0 = 6 \cdot 10^{11} cm^{-2}$)for an input optical power of 20 mW

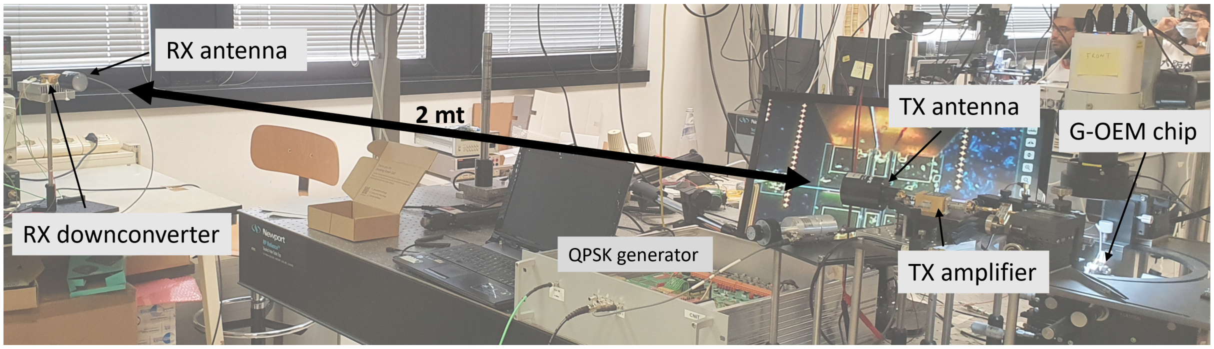

Supplementary Figure 8: wireless link setup, comprising the Treansmitter and receiver sections. At the transmitter, a baseband QPSK signal is generated and connected to the G-OEM chip. This last is visible in the microscope image on the screen. The output of the G-OEM is connected to the TX amplifier. The TX antenna sends the upconverted sub-THz signal to the RX antenna, connected to the commercial RX downconverter.

III. CONVERSION EFFICIENCY VS OPTICAL LOCAL OSCILLATOR POWER

As a complement to the RF characterization presented in the main text, we measured the conversion efficiency as a function of the optical LO power, while keeping a fixed electrical IF input power of 0 dBm. The result is shown in Supplementary Figure 9 .The slope of the experimental curve is 1.9 dB/dB, consistent with the theoretical expected value of (2 dB/dB) that is the usual linear optical power detection regime. Indeed, in the linear regime, the photocurrent is proportional to the optical power, and the associated photo generated electrical power is the square of the photocurrent. Thus, there is a square law between the coupled optical power and the photogenerated electrical power. For 13 dBm OL power, a saturation behavior can be inferred, consistent with Fig. 8b in the main text.

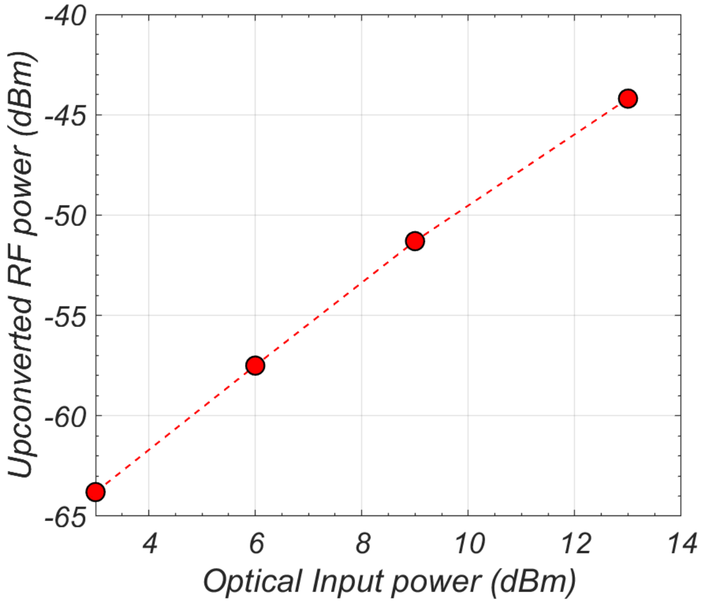

Supplementary Figure 9: G-OEM conversion efficiency vs LO power, for a 0 dBm input IF electrical power.

IV. ERROR-VECTOR MAGNITUDE MEASUREMENT: BACK-TO-BACK

The signal integrity of the starting baseband sequence, that is upconverted and then transmitted through the wireless link, can play a role on the final quality of the received signal. To evaluate its impact, we measured the EVM of the baseband signal generated by the DAC, before being fed to the G-OEM. The measurement, shown in Supplementary Figure 10, effectively reveal a frequency-dependent signal integrity of the starting baseband datastream. In particular, from 1 Gbit/s to 4 Gbit/s the EVM grows from 1.5% up to 5.9%.

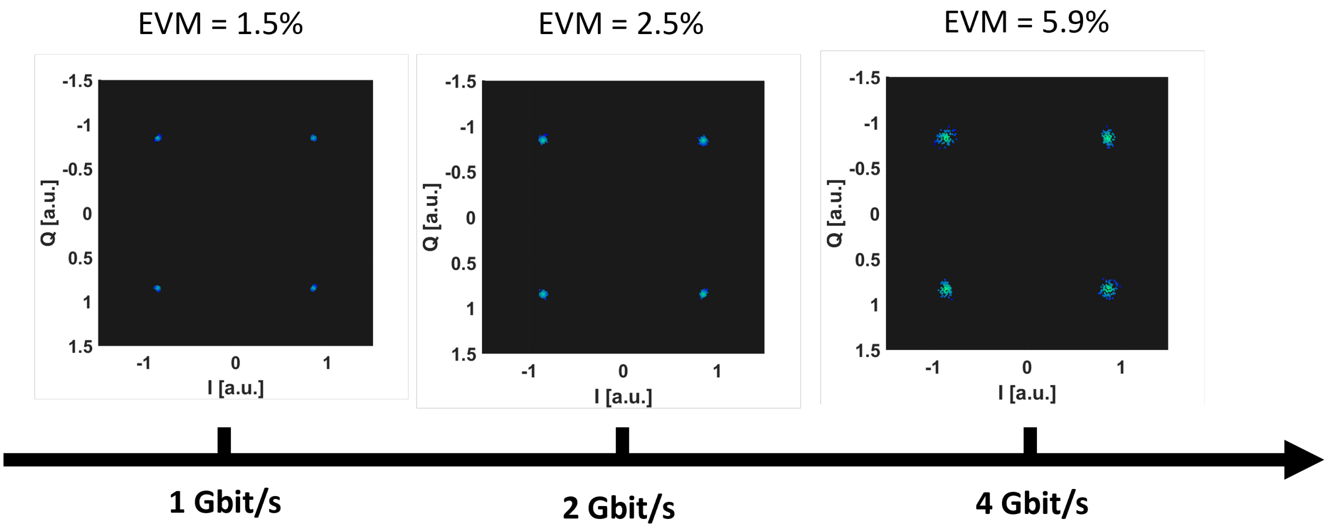

Supplementary Figure 10: QPSK source EVM. Frequency dependence of the EVM of the starting baseband electrical signal generated by the DAC, just before being fed to the G-OEM

V. SUPPLEMENTARY FIGURES

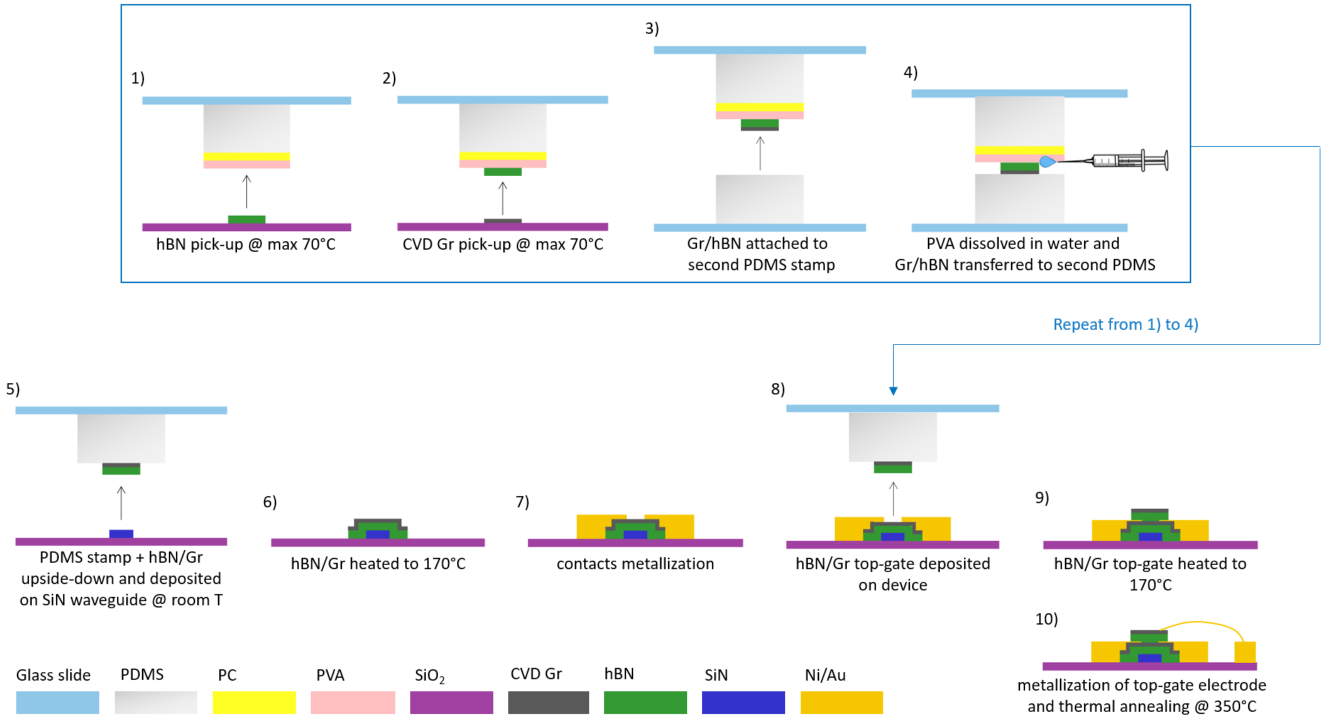

Supplementary Figure 11: G-OEM fabrication flow. The main steps are pick-and-flip of bottom hBN/graphene (from 1 to 6), contact deposition (7), pick-and-flip of top hBN/graphene (from 1 to 4, 8 and 9) and gate contacting (10).

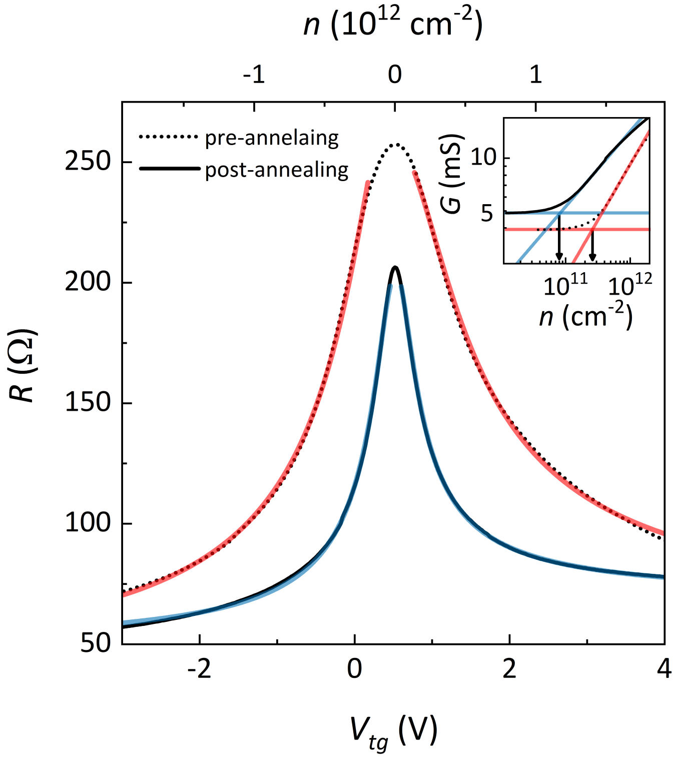

Supplementary Figure 12: Comparison between the resistance of the device as a function of the topgate voltage, measured before (dotted black line) and after (continuous black line) thermal annealing in N2 at 350°C for 3 hours. The light red curves are fits to the pre-annealing data following [23], giving a carrier mobility of $\sim 6000 cm^2 V^{-1} s^{-1}$ for both carrier types. The light blue lines are the same as in the main text, Figure 3e. Inset: comparison between the Log-Log conductance as a function of the carrier density before (dotted black line) and after (continuous black line) thermal annealing. The charge inhomogeneity in the Charge Neutrality Point (CNP) region reduces from $26 \cdot 10^{10} cm^{-2}$ to $8 \cdot 10^{10} cm^{-2}$ upon annealing.

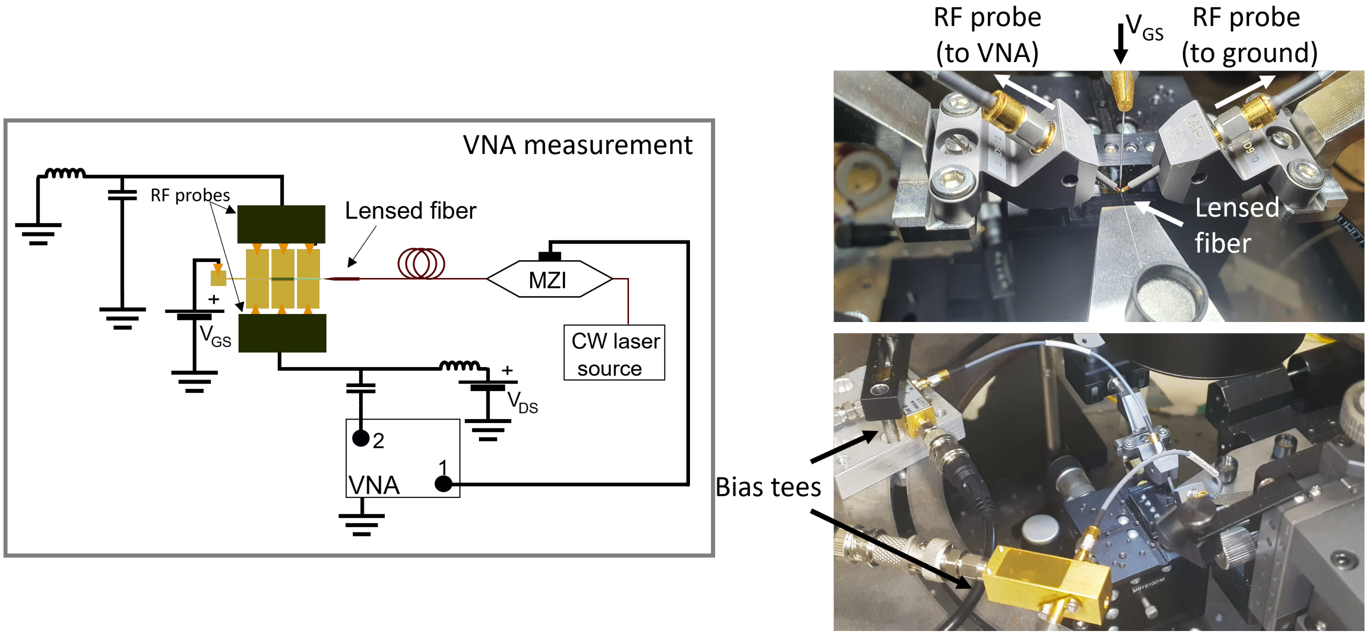

Supplementary Figure 13: Optoelectronic frequency response measurement setup up to 67 GHz. A VNA is used to suurce an RF signal swept from 10 MHz to 67 GHz. This signal drives an MZI modulator which modulates a CW laser. The modulated optical signal is coupled to the G-OEM devices through butt coupling with a tapered fiber. The generated photocurrent from the G-OEM is collected using RF probes, connected to a second port of the VNA. Bias tees are used to decouple the DC and the RF paths.

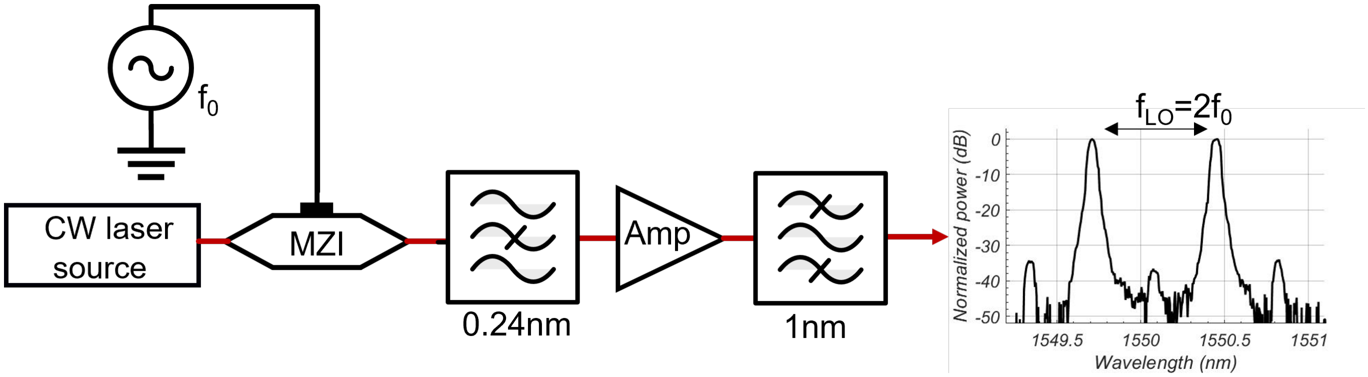

Supplementary Figure 14: Tunable dual wavelength laser source. The optical path is indicated in red, while the black interconnection is electrical. The final output is constituted by two optical tones, separated in frequency by the double of the electrical modulation frequency used to modulate the MZI. The shown optical power is normalized to the peak power of the two optical wavelengths.

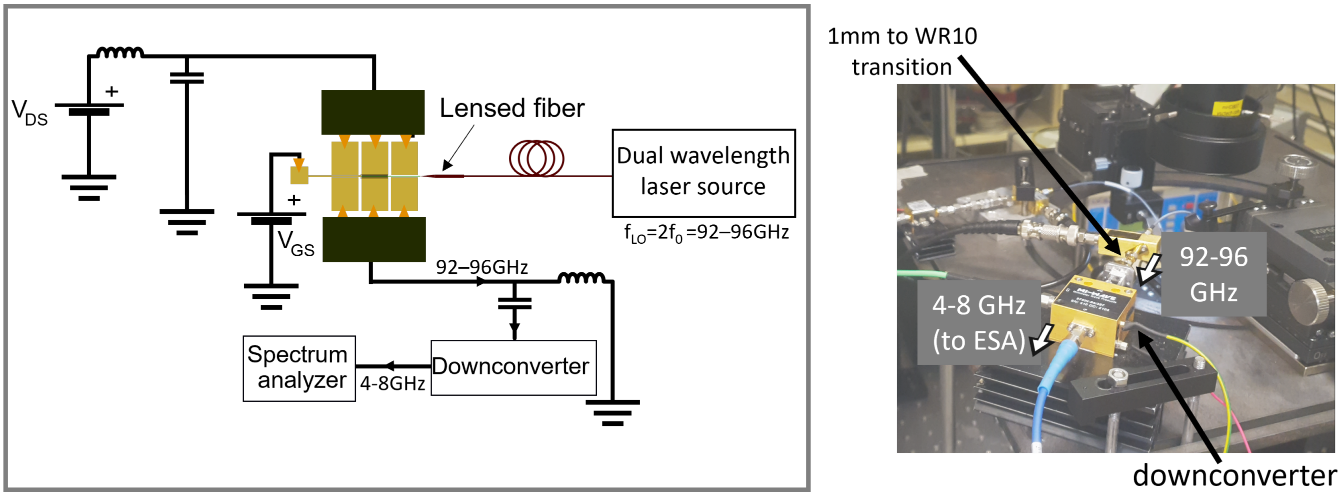

Supplementary Figure 15: Optoelectronic frequency response measurement setup in the 92-96 GHz frequency range. The dual wavelength laser source described in Supplementary Figure 14 is coupled to the G-OEM device using a tapered fiber butt-coupled to the chip. The frequency spacing of the two optical tones composing the dual wavelength source are swept in the range 92-96 GHz. The generated photocurrent from the G-OEM is collected using RF probes, and is fed to a downconverter, which converts the sub-THz electrical signal down to the 4-8 GHz range. The baseband electrical signal is consequently measured using a Spectrum analyzer.

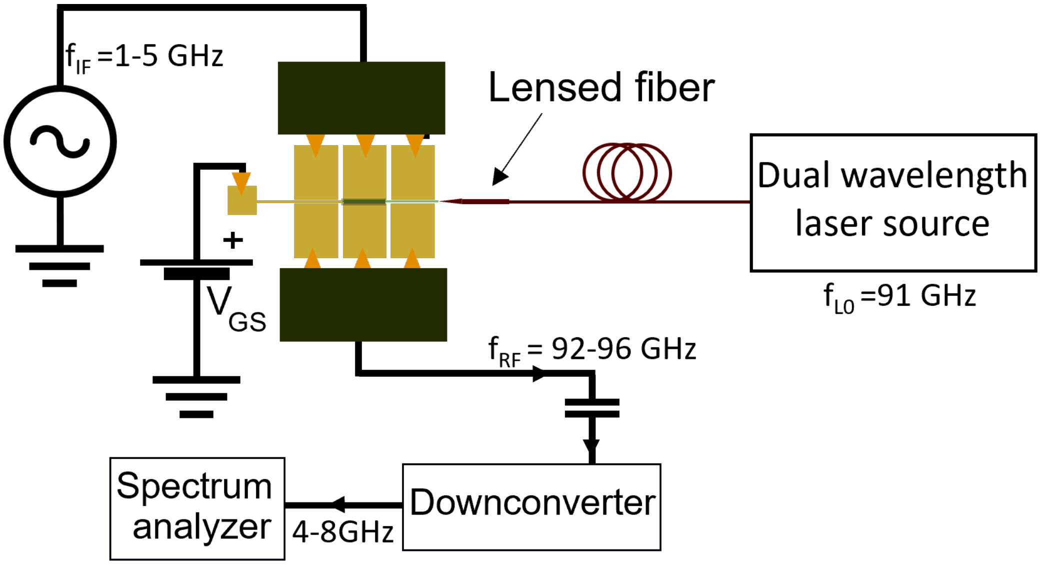

Supplementary Figure 16: Optoelectronic upconversion measurement setup in the 92-96 GHz frequency range. The dual wavelength laser source described in Supplementary Figure 14 is fed to the G-OEM device using a tapered fiber butt-coupled to the chip. The frequency spacing of the two optical tones composing the dual wavelength source is fixed at 91 GHz. Then, an IF electrical sinusoidal signal is applied to one side of the the G-OEM using an RF probe. The resulting mixed signal is upconverted in the frequency range [92/96] GHz = 91 GHz + [1/5] GHz. To measure this upconverted signal, a downconverter is used to convert signal in the range 4-8 GHz. The baseband electrical signal is consequently measured using a Spectrum analyzer.

Supplementary References

[1] Frenzel, A. J., Lui, C. H., Shin, Y. C., Kong, J., and Gedik, N. *Phys. Rev. Lett.* **113**, 056602 Jul (2014).
[2] Koepfli, S. M., Baumann, M., Koyaz, Y., Gadola, R., Güngör, A., Keller, K., Horst, Y., Nashashibi, S., Schwanninger, R., Doderer, M., Passerini, E., Fedoryshyn, Y., and Leuthold, J. *Science* **380**(6650), 1169–1174 (2023).
[3] Marconi, S., Giambra, M. A., Montanaro, A., Mišeikis, V., Soresi, S., Tirelli, S., Galli, P., Buchali, F., Templ, W., Coletti, C., Sorianello, V., and Romagnoli, M. *Nature Communications* **12**(1), 806 (2021).
[4] Ghione, G. *Semiconductor Devices for High-Speed Optoelectronics.* Cambridge University Press, (2009).
[5] Massicotte, M., Soavi, G., Principi, A., and Tielrooij, K.-J. *Nanoscale* **13**, 8376–8411 (2021).
[6] Wang, L., Makk, P., Zihlmann, S., Baumgartner, A., Indolese, D. I., Watanabe, K., Taniguchi, T., and Schönenberger, C. *Phys. Rev. Lett.* **124**, 157701 Apr (2020).
[7] Couto, N. J. G., Costanzo, D., Engels, S., Ki, D.-K., Watanabe, K., Taniguchi, T., Stampfer, C., Guinea, F., and Morpurgo, A. F. *Phys. Rev. X* **4**, 041019 Oct (2014).
[8] Romagnoli, M., Sorianello, V., Midrio, M., Koppens, F. H. L., Huyghebaert, C., Neumaier, D., Galli, P., Templ, W., D'Errico, A., and Ferrari, A. C. *Nature Reviews Materials* **3**(10), 392–414 (2018).
[9] Tomadin, A., Hornett, S. M., Wang, H. I., Alexeev, E. M., Candini, A., Coletti, C., Turchinovich, D., Kläui, M., Bonn, M., Koppens, F. H. L., Hendry, E., Polini, M., and Tielrooij, K.-J. *Science Advances* **4**(5), eaar5313 (2018).
[10] Zebrev, G. I. In *Physics and Applications of Graphene,* Mikhailov, S., editor, chapter 23. IntechOpen, Rijeka (2011).
[11] Sorianello, V., Midrio, M., and Romagnoli, M. *Optics Express* **23**(5), 6478 March (2015).
[12] Principi, A., Lundeberg, M. B., Hesp, N. C., Tielrooij, K.-J., Koppens, F. H., and Polini, M. *Physical Review Letters* **118**(12) March (2017).
[13] Ashcroft, N. W. and Cornell, N. D. *Solid State Physics.* Thomson Press, December (2003).
[14] Tielrooij, K.-J., Hesp, N. C. H., Principi, A., Lundeberg, M. B., Pogna, E. A. A., Banszerus, L., Mics, Z., Massicotte, M., Schmidt, P., Davydovskaya, D., Purdie, D. G., Goykhman, I., Soavi, G., Lombardo, A., Watanabe, K., Taniguchi, T., Bonn, M., Turchinovich, D., Stampfer, C., Ferrari, A. C., Cerullo, G., Polini, M., and Koppens, F. H. L. *Nature Nanotechnology* **13**(1), 41–46 November (2017).
[15] Song, J. C. W., Reizer, M. Y., and Levitov, L. S. *Phys. Rev. Lett.* **109**, 106602 Sep (2012).
[16] Freitag, M., Low, T., Xia, F., and Avouris, P. *Nature Photonics* **7**(1), 53–59 December (2012).
[17] Shi, S.-F., Tang, T.-T., Zeng, B., Ju, L., Zhou, Q., Zettl, A., and Wang, F. *Nano Letters* **14**(3), 1578–1582 February (2014).
[18] Pozar, D. M. *Microwave and RF design of wireless systems.* John Wiley & Sons, Nashville, TN, November (2000).
[19] Giambra, M. A., Mišeikis, V., Pezzini, S., Marconi, S., Montanaro, A., Fabbri, F., Sorianello, V., Ferrari, A. C., Coletti, C., and Romagnoli, M. *ACS Nano* **15**(2), 3171–3187 February (2021).
[20] Ma, P., Salamin, Y., Baeuerle, B., Josten, A., Heni, W., Emboras, A., and Leuthold, J. *ACS Photonics* **6**(1), 154–161 November (2018).
[21] Betz, A. C., Jhang, S. H., Pallecchi, E., Ferreira, R., Fève, G., Berroir, J.-M., and Plaçais, B. *Nature Physics* **9**(2), 109–112 December (2012).
[22] Hamidouche, L., Montanaro, A., Rosticher, M., Grimaldi, E., Poupet, B., Taniguchi, T., Watanabe, K., Plaçais, B., Baudin, E., and Legagneux, P. *ACS Photonics* **8**(1), 369–375 December (2020).
[23] Kim, S., Nah, J., Jo, I., Shahrjerdi, D., Colombo, L., Yao, Z., Tutuc, E., and Banerjee, S. K. *Applied Physics Letters* **94**(6), 062107 (2009).
